# Dynamic Reconstruction Algorithm of Three-Dimensional Temperature Field Measurement by Acoustic Tomography

**DOI:** 10.3390/s17092084

**Published:** 2017-09-12

**Authors:** Yanqiu Li, Shi Liu, Schlaberg H. Inaki

**Affiliations:** Key Laboratory of Condition Monitoring and Control for Power Plant Equipment, Ministry of Education, North China Electric Power University, Beijing 102206, China; liushi@ncepu.edu.cn (S.L.); h.i.schlaberg@ieee.org (S.H.I.)

**Keywords:** acoustic tomography, three-dimensional temperature field, reconstruction algorithm, dynamic model；robust estimation

## Abstract

Accuracy and speed of algorithms play an important role in the reconstruction of temperature field measurements by acoustic tomography. Existing algorithms are based on static models which only consider the measurement information. A dynamic model of three-dimensional temperature reconstruction by acoustic tomography is established in this paper. A dynamic algorithm is proposed considering both acoustic measurement information and the dynamic evolution information of the temperature field. An objective function is built which fuses measurement information and the space constraint of the temperature field with its dynamic evolution information. Robust estimation is used to extend the objective function. The method combines a tunneling algorithm and a local minimization technique to solve the objective function. Numerical simulations show that the image quality and noise immunity of the dynamic reconstruction algorithm are better when compared with static algorithms such as least square method, algebraic reconstruction technique and standard Tikhonov regularization algorithms. An effective method is provided for temperature field reconstruction by acoustic tomography.

## 1. Introduction

Temperature field measurement plays an important role in heat balance calculation, combustion efficiency evaluation, saving energy and reducing pollution. Acoustic temperature field reconstruction is very useful in many real industrial applications because of its advantages, such as non-contact measurement, wide measuring range, ease of real time continuous measurement, convenient maintenance and ease to realize visualization measurement, and so on. Acoustic computed tomography temperature field reconstruction techniques measure the time of flight of sound waves traversing the measured area from multiple directions. An appropriate temperature field reconstruction algorithm is needed to calculate the temperature distribution in the tested area. It belongs to typical inverse problem research, so the development of quick and accurate reconstruction algorithms is critical to realize the temperature field reconstruction. 

Typical acoustic tomography algorithms in temperature field reconstruction can be divided into two-dimensional and three-dimensional reconstruction algorithms. The main algorithms in two dimensional temperature field reconstruction are least square [1,2,3,4,5], Fourier regularization method [6,7,8,9], gaussian function and regularization algorithm [10,11], algebraic reconstruction algorithm [12,13], modified Landweber method [14], Tikhonov regularization method [15,16,17,18,19], radial basis function (RBF) and singular value decomposition [20,21], algorithm based on iteration and interpolation [22], RBF neural network [23], method based on the parabolic function [24], exponent SVD [25,26], exponent regularization [27], genetic algorithm [28], regularization algorithm based on Markov Radial Basis Function [29,30,31], and so on. Two-dimensional temperature field reconstruction can only provide the temperature field distribution of a two-dimensional plane, but the actual measurement object is a three-dimensional space temperature field. It is important to develop a reconstruction algorithm for three-dimensions. Wang et al. [32] performed the acoustic measurement of three-dimensional temperature field by computer analog simulation which constructed acoustic reconstruction algorithm of 3-D temperature field by least squares method, they took cube’s area with 32 acoustic sensors as measuring space which was divided into 64 space grids equally. The spherical temperature field was reconstructed. Wang et al. [33] analyzed the principle of three-dimensional temperature field reconstruction based on acoustic theory and proposed least-square and singular value decomposition (SVD) algorithms for three-dimensional temperature field reconstruction. A three-dimension acoustic pyrometry model is established to describe the relationship between the time of flight and speed of the sound in a cubic space. The Least square method and singular value decomposition algorithms are effective and widely used in many fields and are applied to the reconstruction of three-dimensional temperature fields. Three typical temperature field models, such as the one-peak model, two-peak model and four-peak model, were simulated with only a few acoustic data by the least squares method and SVD algorithm. An et al. [34] proposed Conjugate Gradient Least Squares (CGLS) and Least Squares QR-Decomposition (LSQR) for three-dimensional temperature field reconstruction. A mathematical model was applied based on acoustic theory. Conjugate gradient least squares and least squares QR-Decomposition methods are used to solve a large ill-posed matrix equation. Zhou et al. [35] presented an algorithm based on radical basis function (RBF) neural networks to reconstruct the three-dimensional temperature field. The algorithm used the three-dimensional discrete cosine transform (DCT) on temperature fields and established a mapping relation between the low order term coefficient vector and the sound wave path average temperature vector, then implemented the mapping relation using a radical basis function (RBF) neural network that has a strong function fitting ability. The three-dimensional temperature field was reconstructed by using the inverse three-dimensional discrete cosine transform. An et al. [36] proposed two computer tomography algorithms, including the algebraic reconstruction technique (ART) and simultaneous iterative reconstruction technique (SIRT); with these, three typical temperature field models were reconstructed for flame distribution in a boiler furnace, namely the single-peak model, double-peak model and four-peak model. Subsequently the simulation results between the above two algorithms were compared and their anti-noise capability was analyzed. Two regularization algorithms based on the singular value decomposition algorithm are proposed in [37]. Three typical temperature fields were simulated. The Anti-noise ability of the algorithms was tested using Gaussian noise with standard deviations. An et al. [38] developed the Generalized minimum residual method (GMRES) which was suitable for solving large sparse matrix equations. GMRES is applied to reconstruct the three-dimensional temperature field in the boiler furnace. Numerical simulation is made in two model temperature field that are single-peaked symmetry and single-peaked deflection by MATLAB. Manuela Barth et al. [39] developed an acoustic tomographic measurement system which is capable of resolving three-dimensional distributions of temperature and flow fields in air within a certain volume (1.3 m × 1.0 m × 1.2 m) using 16 acoustic transmitter–receiver pairs. Algorithms for the 3D reconstruction of distributions from line-integrated measurements are presented. SIRT algorithm is adopted to compute temperature distribution in their calculations. A linkage between adjacent grid cells is implemented which accounts for the assumption that the distributions are continuous fields. Moreover, a measuring apparatus is introduced which is suited for demonstration of the method as well as for indoor investigations. Example temperature measurements without any flow within a laboratory using this system are shown. Three-dimensional temperature distribution slice maps in two moments are shown. Furthermore, alternative individual measurement methods for temperature and flow speed provide comparable results.

The essence of acoustic tomography algorithms in temperature field reconstruction is to obtain the temperature field distribution by optimization technique. The algorithms in temperature field reconstruction can be broadly classified into three categories according to the reconstruction principle; they are the non-iterative algorithm, iterative algorithm and intelligent optimization algorithm. The non-iterative reconstruction algorithms include least square method, singular value decomposition, Tikhonov regularization method, Fourier regularization method, gaussian function, regularization algorithm, and so on. The iterative algorithms contain ART and SIRT, Landweber method, CGLS, LSQR, and so on. The intelligent optimization algorithms include the genetic algorithm, the RBF neural network, and so on. The non-iterative algorithms are simple to achieve, fast to calculate with low accuracy. The iterative algorithms and intelligent optimization algorithms have higher accuracy with longer computation time compared with the non-iterative algorithms.

In general, the algorithms mentioned above played an important role in the development of acoustic temperature measurement technology and its successful application. But the time dynamic information in the dynamic change process of the temperature field is not considered in these algorithms as they are all static reconstruction algorithms. A practical temperature field measured by acoustic tomography is an object that is changing over time. Traditional static reconstruction algorithms only consider the measurement information of the acoustic measurement, not focusing on the dynamic information of the dynamic processes in the temperature field. It is shown that there is close correlation between the temperature field images in the dynamic process at different times [40,41]. It is more reasonable to use a dynamic imaging reconstruction algorithm for a dynamic object. An objective function is proposed to simultaneously consider the acoustic measurement information, the dynamic development information of the dynamic object, plus space constraints. The robust estimation is adopted to extend the objective function. In addition, the temperature field reconstruction is often represented as an optimization problem, thus it is very important to research for an actual effective optimization algorithm. The tunneling algorithm [42] is adopted to solve the objective function. Feasibility of the proposed dynamic reconstruction algorithm is verified through simulation.

The remainder of this paper is structured as follows: Section 2 discusses the principle of acoustic pyrometry and the static model. Section 3 establishes the dynamic reconstruction model of three-dimensional temperature field measurement and solves the objective function. The details of temperature field reconstruction using measurement signals without noise are presented in Section 4. Temperature field reconstruction using measurement signals with noise are described in Section 5. The conclusions are presented in Section 6.

## 2. The Principle of Acoustic Pyrometry and the Static Model

The principle of acoustic pyrometry is based on that the propagation speed of sound in a gas medium is a function of the gas temperature.

(1) $$v=\sqrt{\frac{\gamma RT}{m}}=Z\sqrt{T}$$

where *v* is the propagation speed of the acoustic wave in a gas medium, m/s; *R* is the molar gas constant, J/mol·k; *γ* is the gas specific heat ratio, *T* is the absolute temperature of gas, K, *m* is the molar mass of the gas, kg/mol, *Z* = $\sqrt{\gamma R/M}$, it is a specific constant of the gas.

The principle of acoustic tomography temperature measurement is to solve a three-dimensional temperature field distribution based on the time of flight (TOF) of multiple paths. After obtaining the TOF of each path and the discretization of the temperature field, algebraic equations are established to represent the relationship between the temperature in discrete grids and the TOF. The problem of temperature field reconstruction is converted to a problem of solving equations. 

It is assumed that there are M acoustic paths in the acoustic temperature measurement system, where the three-dimensional furnace space is divided into N units. The TOF is represented by:

(2) $$y_{i}=\int_{L_{i}}\frac{1}{v_{j}(x,y,z)}dl+n_{i}=\int_{L_{i}}f_{j}(x,y,z)dl+n_{i}$$

in which, *y_i_* represents the time of flight of the wave along the ith sound path; *L_i_* is the *i*th sound wave ray transmission path; (*x,y,z*) is the location of the unit, *v_j_*(*x,y,z*) is the sound speed of the *j*th imaging unit; *f_j_*(*x,y,z*) is the slowness of the jth pixel units (i.e., the reciprocal of velocity), *n_i_* is the measurement noise. An equation set is obtained after one measurement cycle. Formula (2) can be simplified as a static reconstruction model in the form of a matrix equation:

(3) Y=AF+n

where *A* $\in R^{M\times N}$ represents the line segment length that the *j*th pixel is cut out by *i*th rays, $Y\in R^{M}$ is the TOF vector measured in practice, $F\in R^{N}$ represents the space state factor, i.e., the reciprocal of velocity. *M* stands for the total of independent TOF measurements across the temperature field, *N* is the number of units into which the reconstruction space is divided, $n\in R^{M}$ represents the noise vector in the TOF measurement data. F is calculated by a reconstruction algorithm, and then the temperature *T*(*x,y,z*) is obtained according to (4).

(4) $$T(x,y,z)=\frac{1}{F{\left(x,y,z\right)}^{2}Z^{2}}$$

## 3. Modeling and Solving of the Dynamic Reconstruction Model

### 3.1. The Establishment of the Dynamic Reconstruction Model

Only the acoustic measurement information of the time of flight is considered in the static temperature field reconstruction model. But the dynamic evolution of the temperature field information is not taken into account. Studies have shown that the reconstruction quality can be improved by increasing the amount of the acoustic temperature field reconstruction information [10]. For temperature field measurements, a direct way to increase the amount of information is to utilize both the acoustic temperature field measurement information and the dynamic development information at the same time. Therefore, the dynamic reconstruction model can be expressed as

(5) $$F_{k}=g(F_{k-1},w_{k})$$

(6) $$y_{k}=h(F_{k},u_{k})$$

where *F_k_* represents the slowness variable at time k; *g*(·) describes the dynamic development information expressed by a series of partial differential equations in the temperature field measurement; *h*(·) is a measurement equation; *y_k_* represents the TOF value at moment k; *w_k_* and *u_k_* represents the uncertainty in the dynamics equation and measurement equation respectively; and the subscript *k* is the index of the discrete time. Formulas (5) and (6) can be approximated to linear equations in order to realize a rapid reconstruction

(7) $$F_{k}=B_{k}F_{k-1}+w_{k}$$

(8) $$y_{k}=A_{k}F_{k}+u_{k}$$

where *B_k_* is the state transition matrix at time k; *A_k_* is measurement operator. If *B_k_* = *I*, *I* is a unit matrix. Formula (7) can be regarded as a pure random walk evolution model which is usually adopted in practice when no better dynamic model is known [43].

It is difficult to solve Equations (7) and (8) directly so they are transferred to an optimization problem. The optimization problem is expressed as Equation (9) according to Tikhonov regularization theory and optimization theory.

(9) $$\underset{F_{k}}{\min}Z={\left\|y_{k}-A_{k}F_{k}\right\|}^{2}+\lambda_{1}{\left\|D_{k}F_{k}\right\|}^{2}+\lambda_{2}{\left\|F_{k}-B_{k}F_{k-1}\right\|}^{2}$$

*λ*_1_ and *λ*_2_ are non-negative regularization factors, ${\left\|DF_{k}\right\|}^{2}$ and *D* are the regularization term and regularization matrix respectively which function to balance the accuracy and stability of the solution.

**Figure 1:**
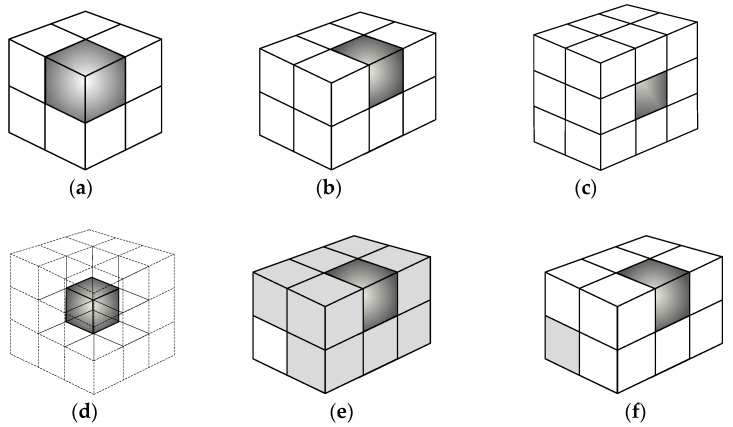
The adjacent relation between space pixels. (**a**) The space pixels are located on the eight corners; (**b**) The space pixel is located in the middle of the side boundary; (**c**) The space pixel is located in the center of the plane; (**d**) The space pixel is located in the center of the cubic; (**e**) The space pixels are adjacent to the boundary of the *i*th space pixel; (**f**) The space pixels are adjacent to the vertex of the *i*th space pixel.

The regularization matrix *D* can be chosen according to different research objects. Different regularization methods are obtained depending on the choice of different matrix *D*. The smooth constraint method is adopted to impose smooth constrain as prior information to solve ill-posed inverse problem that makes the parameters between adjacent grid cells smooth [44,45,46]. A regularization operator *D* that is suitable for three-dimensional temperature field reconstruction is established based on the smooth constraint method according to the continuous distribution feature of three-dimensional temperature field.

(10) $$D_{i,j}=\left\{ \begin{array}{l} 1\;\mathrm{if}\;j=i\\ \frac{1}{p}\;\mathrm{if}\;j\in \Omega_{i}\\ \frac{1}{p}\;\mathrm{if}\;j\in \Phi_{i}\\ 0\;\mathrm{others} \end{array}\right.\;(1\leq i,j\leq N)$$

where Ω*_i_* is the pixel sets that are adjacent to the boundary of *i*th space pixel; Φ*_i_* is the pixel sets that are adjacent to the vertex of *i*th space pixel. The relationship between a space pixel and its adjacent space pixel can be classified into four conditions (shown in Figure 1), because the distribution of three-dimensional temperature field is continuous in the measured area. It is shown in Figure 1a when the space pixels are located on the eight corners; it is shown in Figure 1b when the space pixel is located in the middle of the side boundary; it is shown in Figure 1c when the space pixel is located in the center of the plane; it is shown in Figure 1d when the space pixel is located in the center of the cubic; it is shown in Figure 1e when the space pixel are adjacent to the boundary of the *i*th space pixel; it is shown in Figure 1f when the space pixels are adjacent to the vertex of the *i*th space pixel. The *N* × *N* regularization matrix *D* is obtained in turn in which *p* is the sum of the pixel sets that are adjacent to the *i*th space pixel and *N* is the sum of the space pixels.

### 3.2. Extension of Objective Function by Using Robust Estimation

The Sum of squares function is adopted to measure the accuracy of measurement data in (9). Applications show that estimations by least squares are seriously affected by an abnormal value in the dataset [47]. A stable estimation method should be used in practice. Robust estimation was proposed, aiming at improving the poor anti-interference performance of least square method to gross error [48]. A kind of estimation method is constructed to obtain the optimum estimate with strong resistance against gross error. Maximum likelihood-type estimation (M-estimation) is a kind of robust estimation that is applied widely and has many successful applications [49,50,51,52,53]. The function of the residual error is used to replace squared residuals in M-estimation, the equation is as follows

(11) $$\min H=\sum_{i=1}^{I}\phi (r_{i})$$

where ϕ(·) is M-estimation function, $r_{i}=A_{i}F-Y_{i}$, *I* represents the amount of TOF measurement data. M-estimation functions usually used consist of: absolute value function, Huber function, Talvar function, Fair function, Cathy function, G-M function and Hampel function. The Least square estimation is replaced with M-estimation in (9)

(12) $$\underset{F_{k}}{\min}J=\sum_{i=1}^{I}\varphi (r_{i})+\lambda_{1}{\left\|D_{k}F_{k}\right\|}^{2}+\lambda_{2}{\left\|F_{k}-B_{k}F_{k-1}\right\|}^{2}$$

The Cauchy function is chosen as the M-estimation for convenience in this paper, therefore, a cost functional containing the M-estimation can be written as

(13) $$\underset{F_{k}}{\min}J=\sum_{i=1}^{I}\left(\frac{\beta^{2}}{2}\ln(1+{\left(\frac{r_{i}}{\beta }\right)}^{2}\right))+\lambda_{1}{\left\|D_{k}F_{k}\right\|}^{2}+\lambda_{2}{\left\|F_{k}-B_{k}F_{k-1}\right\|}^{2}$$

The choice of the regularization parameter plays an important role in the accuracy of the reconstruction results when solving the inversion problem, which is always a challenge when dealing with ill-posed problems. There is no one universal method for choosing the regularization parameter when solving ill-posed problems. The choice of regularization parameters *λ*_1_ and *λ*_2_ is a problem of choosing multiple regularization parameters which is commonly solved using a L-hyperplane [54]. The L hyper plane is considered as the multi-dimensional extension of a typical L curve method that is a curve representing the residual norm and constrained norm with a proper scale. Intuitively, the “generalized corner” of a L-hyperplane should be the approximate balance point between the regularization error and the disturbance error. The main disadvantage of the L-hyperplane method is the high computation cost of estimating the maximum of the Gaussian curvature for a large number of regularization parameters. Moreover, positioning the maximum of the Gaussian curvature by regular optimization techniques is limited by the fact that there are multiple extrema in the Gaussian curvature function. 

The minimum distance function (MDF) method [55] is proposed to consider replacing the Gaussian curvature by a surrogate minimum distance function (MDF) which is far easier to optimize. 

It is shown that the formulation of the problem leads naturally to an efficient fixed-point iteration to determine the optimal parameters. Simulation experiments demonstrate that there is little performance loss incurred through the optimization of the MDF as opposed to direct maximization of the Gaussian curvature; however, the computational burden is significantly smaller. The MDF method is adopted to determine the regularization parameters in this paper to make sure the regularization parameters obtained by the function optimization are close to those of the L-hyperplane. 

Let O = (*a*,*b*_1_,*b*_2_) represent the coordinates of the origin. The MDF *ν*(**λ**) is the distance from the origin to the point on the L-hyperplane.

(14) $$\nu (\lambda )={\left|\psi [z(\lambda )]-a\right|}^{2}+\sum_{i=1}^{2}{\left|\psi [x_{i}(\lambda )]-b_{i}\right|}^{2}$$

where

(15) ψ(t)=log(t)

(16) $$z(\lambda )={\left\|Y-AF^{*}(\lambda )\right\|}_{2}^{2}$$

(17) $$x_{i}(\lambda )=\Phi_{i}[R_{i}F^{*}(\lambda )],\;i=1,2$$

in which the definition of ***λ*** and F^*^ is as follow

(18) $$F^{*}(\lambda )=\underset{F_{k}}{\arg\min}\{\sum_{i=1}^{I}\left(\frac{\beta^{2}}{2}\ln(1+{\left(\frac{r_{i}}{\beta }\right)}^{2}\right))+\lambda_{1}{\left\|D_{k}F_{k}\right\|}^{2}+\lambda_{2}{\left\|F_{k}-B_{k}F_{k-1}\right\|}^{2}\}$$

The Minimum distance point is the point where the Gaussian curvature of the L-hyperplane is positive and *ν*(*λ*) reaches a local minimum.

(19) $$\lambda^{*}=\underset{\lambda \in R^{M}}{\arg\min}\nu (\lambda )$$

λ^*^ can usually be found by any optimization technique, but many optimization algorithms require calculating the high-order derivative of *z*(*λ*) and *x_i_*(λ) with respect to *λ_i_* and these derivatives are obtained by solving a linear system whose size is the same as that of the original problem and are calculated in sequence from $\frac{\partial F^{*}(\lambda )}{\partial \lambda_{i}}$. For ease of calculation, a fixed-point algorithm for λ^*^ is obtained using the basic characteristics of the MDF.

(20) $$\lambda_{i}^{\left(k+1\right)}=\frac{z(\lambda^{\left(k\right)})}{x_{i}(\lambda^{\left(k\right)})}\left(\frac{\log[x_{i}(\lambda^{\left(k\right)})]-b_{i}}{\log[z(\lambda^{\left(k\right)})]-a}\right),i=1,2$$

where $\lambda^{\left(k\right)}$ is a vector of the regularization parameters at step *k*. The algorithm is started with an appropriate initial regularization parameter vector $\lambda^{\left(0\right)}$ and the stop criteria is reached when the relative change of the iteration is less than 10^−4^.

### 3.3. Solving of the Objective Function

Solving (13) plays a critical role in the successful application of the dynamic reconstruction method. The tunneling algorithm is introduced to design an effective iteration scheme for searching a possible optimal global solution. The tunneling algorithm was first proposed by Levy and Montalvo [42], which was an effective deterministic algorithm for global optimization. It is an important research direction because of its advantages of fast optimization searching and fine optimization effect. A tunneling function is constructed in local minimum and the local minimum is jumped out of by minimizing the tunneling the function to reach another local minimum less than the function value. The calculation process is carried out repeatedly until the global minimum point is sought out. The tunneling algorithm provides a method to solve global optimization by the local optimization tool and shows great superiority in many applications of science subjects and engineering field [56,57,58].

The tunneling algorithm consists of two phases: minimization phase and tunneling phase. The global minimization of the objective function Z(F) is found by using these two phases alternatively. In the minimization phase, a random start point *F*_0_ is given to find a local minimum F* of the objective function Z(F) by a classical nonlinear programming algorithm.

In the tunneling phase, an auxiliary function $P(F,F^{*})$ is defined called a tunneling function which is a first order continuous differentiable function whose zero-set coincides with the set where $Z(F)=Z(F^{*})$. The aim of this phase is to search a new point *F*_0_ to satisfy $P(F_{0},F^{*})$ ≤ 0 starting from any point in the neighborhood of F*. For this purpose, any classical nonlinear programming algorithm is used to minimize $P(F_{0},F^{*})$ in the tunneling phase. The function value of the minimization series is not greater than that of previous minimizations by carrying out these two phases alternatively, that is, the function value in the local minimization is decreasing. It is obvious that the design of the tunneling function is very important in the application. These are some commonly used tunneling functions and modified tunneling functions [42,59].

(21) $$P_{1}(F,F^{*})=\frac{Z(F)-Z(F^{*})}{{\left[{\left(F-F^{*}\right)}^{T}(F-F^{*})\right]}^{\alpha }}$$

(22) $$P_{2}(F,F^{*})=\left\|F-F_{0}\right\|\frac{{\left(Z(F)-Z(F^{*})+r\right)}^{2}}{1+q{\left(Z(F)-Z(F^{*})+r\right)}^{2}}$$

(23) $$P_{3}(F,F^{*})=\frac{1}{\left\|F-F_{0}\right\|}\frac{{\left(Z(F)-Z(F^{*})+r\right)}^{2}}{1+q{\left(Z(F)-Z(F^{*})+r\right)}^{2}}$$

where α is the intensity of ${\left(F-F^{*}\right)}^{T}(F-F^{*})，\;F_{0}\notin \Omega$, *q* > 0 and *r* satisfies $0<r<\max(Z(F^{*})-Z(F_{1}^{*}))$.

In the tunneling phase, there is a new starting point $F_{0}$ satisfying $Z(F_{0})<Z(F^{*})$, if $F^{*}$ is not the global minimum of J(F), $F_{0}$ is served as a new initial point to minimize the objective function Z(F). In practice, it is not necessary to find the minimum value of $P(F,F^{*})$ in the tunneling algorithm. In the case of point F being found meeting $P(F)<P(F^{*})$, the algorithm turns to the minimization phase so that a better minimum of the objective function can be found with any classical nonlinear programming algorithm.

## 4. Temperature Field Reconstruction with Noiseless Measurement Signals 

**Figure 2:**
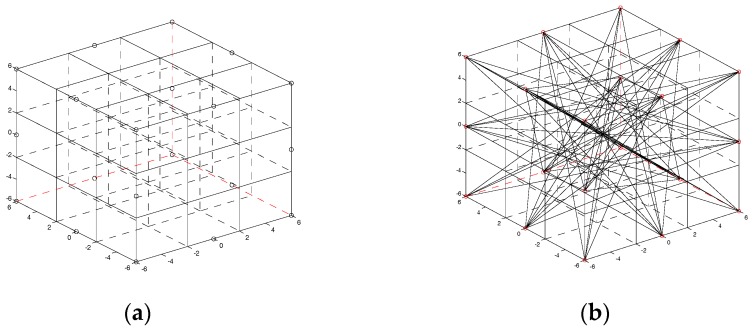
Illustration of the transducer arrangement and the rays. (**a**) Transducers arrangement; (**b**) Sound rays between transducers.

In this part, a numerical simulation is adopted to verify the feasibility of the dynamic reconstruction algorithm based on robust estimation. The reconstruction quality is compared with that of the least square method (LSM), algebra reconstruction technique (ART), simultaneous iterative reconstruction technique (SIRT) and standard Tikhonov (STR) regularization method. A cube space of 12 m × 12 m × 12 m is chosen and 20 acoustic sensors are arranged in three different planes respectively, as shown in Figure 2. Acoustic source signal is chosen which frequency is 1.5–10 KHz and sound pressure level is greater than 126 dB because the combustion noise in the boiler mainly focuses on the low-frequency stage and the high-frequency acoustic wave has large attenuation. The whole image reconstruction domain is divided into 27 subdomains (3 × 3 × 3). A bicubic interpolation with 31 × 31 × 31 items are adopted after getting the temperature in each subdomain. The simulation test is conducted on a computer with an Intel(R) i7-7500, 2.9GHzCPU, 8G memory and MATLAB2016a as the development tool.

**Figure 3:**
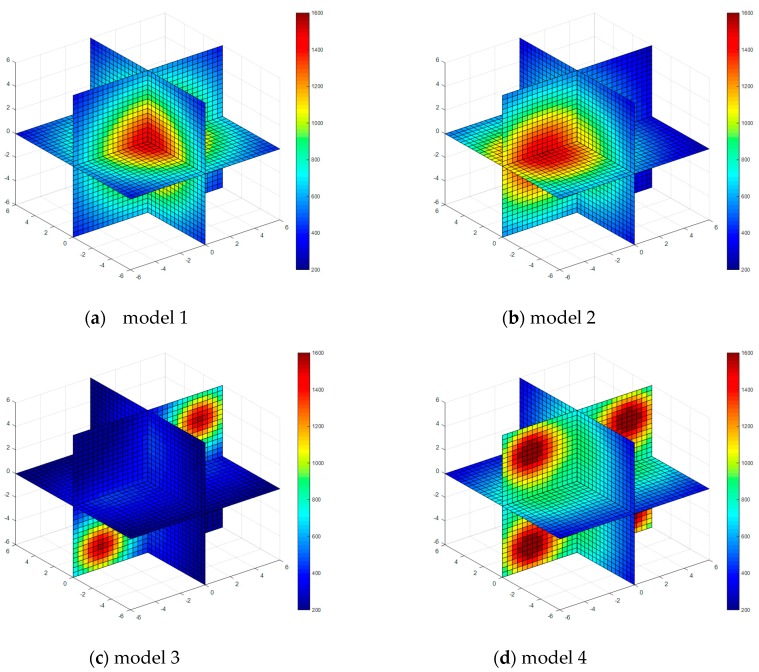
Diagrams of model temperature fields.

Four kinds of classical temperature fields are chosen for simulation, these are: model 1 (single-peak symmetric), model 2 (single-peak deflection), model 3 (double peak symmetric) and model 4 (four peaks symmetric), the temperature field model distributions are shown in Figure 3.

The single-peak symmetric model temperature field is

(24) $$T=\frac{1500}{0.04(x^{2}+y^{2}+z^{2})+1}$$

The single-peak deflection model temperature field is

(25) $$T=\frac{1500}{0.04({\left(x+3\right)}^{2}+y^{2}+z^{2})+1}$$

The double-peak symmetric model temperature field is

(26) $$\begin{array}{l} T=\frac{1500}{0.2({\left(x-4\right)}^{2}+y^{2}+{\left(z-4\right)}^{2})+1}+\\ \frac{1500}{0.2({\left(x+4\right)}^{2}+y^{2}+{\left(z+4\right)}^{2})+1} \end{array}$$

The four-peak symmetric model temperature field is

(27) $$\begin{array}{l} T=\frac{1500}{0.2({\left(x+4\right)}^{2}+y^{2}+{\left(z+4\right)}^{2})+1}+\\ \frac{1500}{0.2({\left(x+4\right)}^{2}+y^{2}+{\left(z-4\right)}^{2})+1}+\\ \frac{1500}{0.2({\left(x-4\right)}^{2}+y^{2}+{\left(z+4\right)}^{2})+1}+\\ \frac{1500}{0.2({\left(x-4\right)}^{2}+y^{2}+{\left(z-4\right)}^{2})+1} \end{array}$$

The time delay Y of the signals, that is, time of flight (TOF), is estimated by line integral Equation (28).

(28) $$Y_{k}=\int_{L_{k}}1/Z\sqrt{T(x,y,z)}ds$$

where *Y_k_* is the time delay of the *k*th acoustic ray, *L_k_* is the sound wave path of the *k*th sound ray.

**Figure 4:**
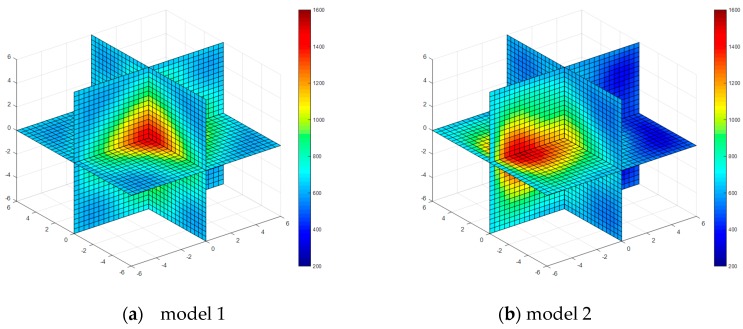

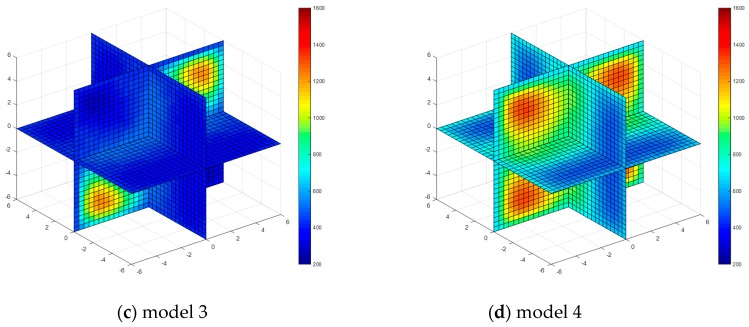
Diagram of reconstructed temperature fields by the least squares method.

**Figure 5:**
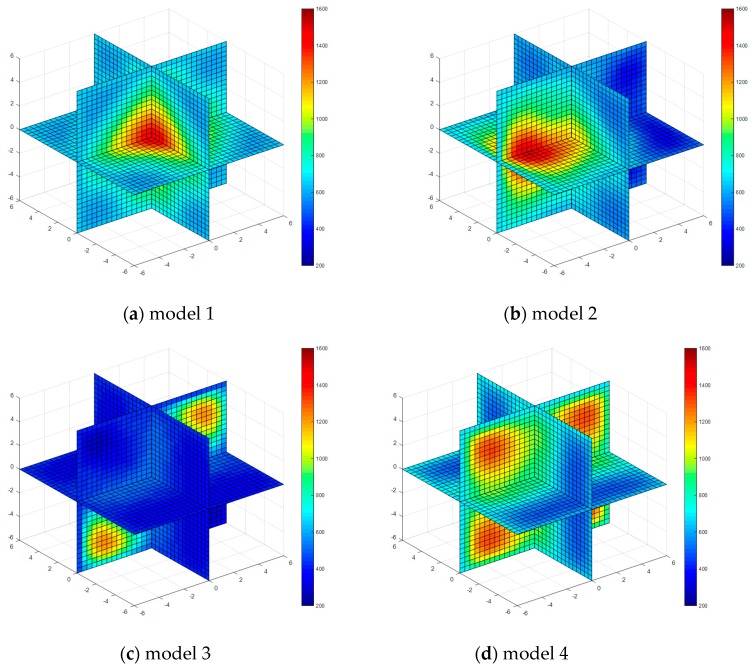
Diagram of reconstructed temperature fields by algebraic reconstruction technique (ART).

**Figure 6:**
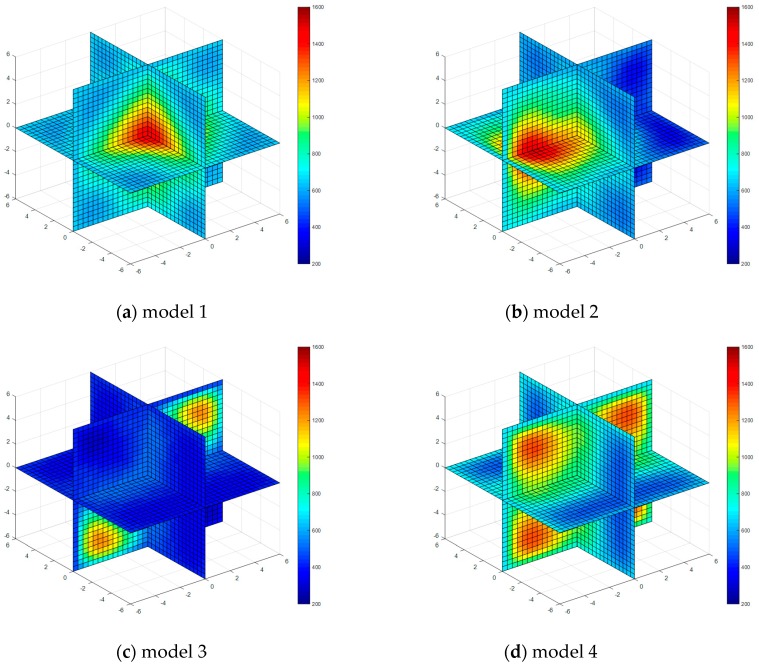
Diagram of reconstructed temperature fields by standard Tikhonov regularization method.

**Figure 7:**
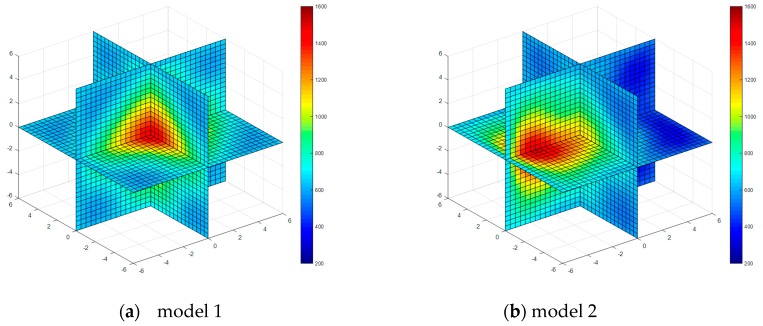

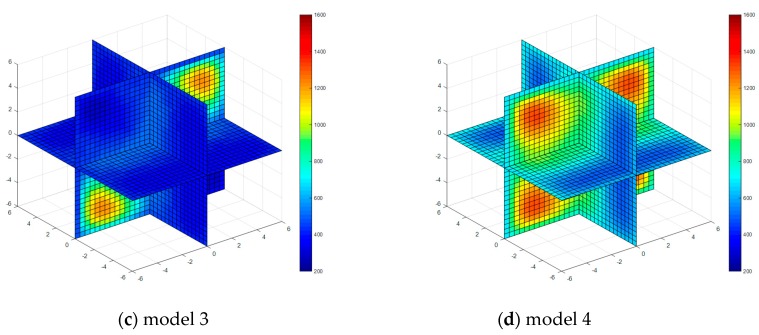
Diagram of reconstructed temperature fields by SIRT.

**Figure 8:**
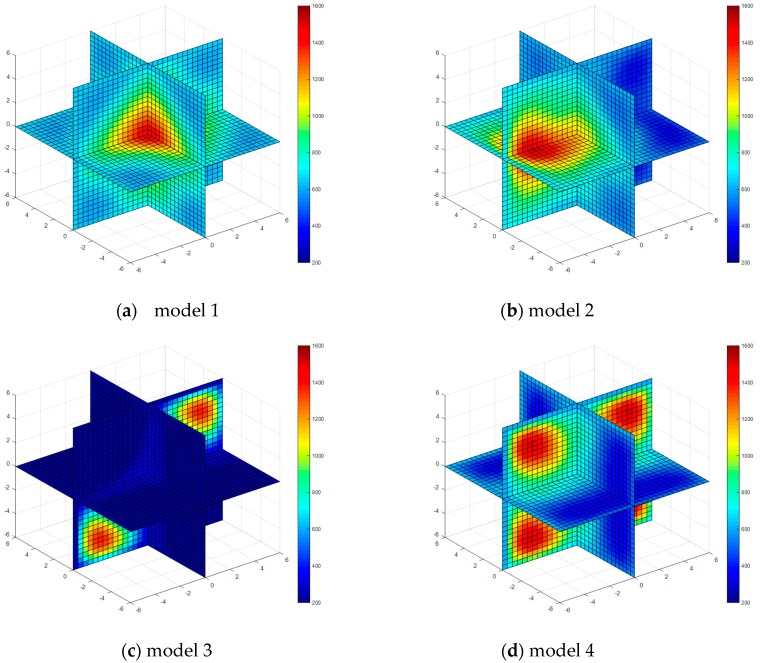
Diagram of reconstructed temperature fields by dynamic reconstruction algorithm.

The temperature field reconstruction results from the least square method, algebraic reconstruction technique, standard Tikhonov Regularization, simultaneous iterative reconstruction technique and dynamic reconstruction algorithm based on robust estimation (DRARE) are shown in Figure 4, Figure 5, Figure 6, Figure 7 and Figure 8. The regularization property in the standard Tikhonov regularization algorithm is chosen as 0.00001. The parameters chosen for ART are shown in Table 1. The iteration number of SIRT is 216, the iteration is terminated when the norm of the difference between the last two iteration results is less than 10^−3^. The images of the reconstruction temperature fields show that the five algorithms can reconstruct images confirming the model temperature fields.

Three kinds of errors, namely the maximum relative error $E_{1}$, the average relative error $E_{2}$ and the root mean square error $E_{3}$ are adopted to evaluate the reconstruction results of the five algorithms. The errors are defined as (29)–(31) respectively.

(29) $$E_{1}=\frac{\left|T_{M\max}-T_{R\max}\right|}{T_{M\max}}\times 100\%$$

(30) $$E_{2}=\frac{\left|T_{Ma}-T_{Ra}\right|}{T_{Ma}}\times 100\%$$

(31) $$E_{3}=\frac{\sqrt{\frac{1}{N}\sum_{j=1}^{N}{\left[T_{M}(j)-T_{R}(j)\right]}^{2}}}{T_{M\max}}\times 100\%$$

where *N* is the pixel count of the reconstructed temperature field, *T_M_*(*j*) is the temperature value of the model temperature field, *T_Ma_* is the average temperature of the model temperature field, *T_Mmax_* is the maximum of the model temperature field, *T_R_*_(*j*)_ is the temperature value of reconstructed temperature field, *T_Ra_* is the average temperature of the reconstructed temperature field, *T_Rmax_* is the maximum of the reconstructed temperature field.

The reconstruction errors of the four algorithms are shown in Table 2. The reconstruction errors of LSM are close to those of STR, because the improvement of STR in an overdetermined problem is limited. The reconstruction results of ART and SIRT are slightly better than those of LSM and STR, and the results of DRARE are significantly improved. In the temperature field of the single-peak symmetric example, the reconstruction time with LSM is 0.57 s, the reconstruction time with ART is 0.83 s, the reconstruction time with STR is 0.61 s, the reconstruction time with SIRT is 0.92 s, the reconstruction time with DRARE is 0.76 s. The reconstruction time with the five algorithms are similar, but the errors of DRARE is the smallest.

The convergence and accuracy of the algorithm with different number transducers are analyzed. Six cases with different number transducers and sound rays, that is, 22 transducers (72 sound rays), 21 transducers (65 sound rays), 20 transducers (58 sound rays), 19 transducers (51 sound rays), 18 transducers (45 sound rays) and 17 transducers (38 sound rays), are chosen to analyze the performance of the algorithm. The simulation results show that the algorithm is convergent in the cases of 72, 65, 58, 51 and 45 sound rays and the accuracy reduces with the decreasing number of the transducers. The algorithm cannot be converged to an efficient solution when the sound rays are reduced to 38 rays, because condition number of coefficient matrix of normal equation reaches 4 × 10^16^ and it is seriously ill-posed.

The performance of the algorithm is compared with that of the static reconstruction algorithm (the least square) when more measurement points (22 transducers) and fewer measurement points (18 transducers) are used. Single-peak symmetric model temperature field is taken as an example to compare the reconstruction accuracy. The root mean square errors of the least square method are 1.64% in the case of 22 transducers and 3.35% in the case of 18 transducers respectively. The root mean square errors of DRBRA are 0.23% in the case of 22 transducers and 0.61% in the case of 18 transducers respectively.

## 5. Temperature Field Reconstruction Using Measurement Signals with Noise

**Figure 9:**
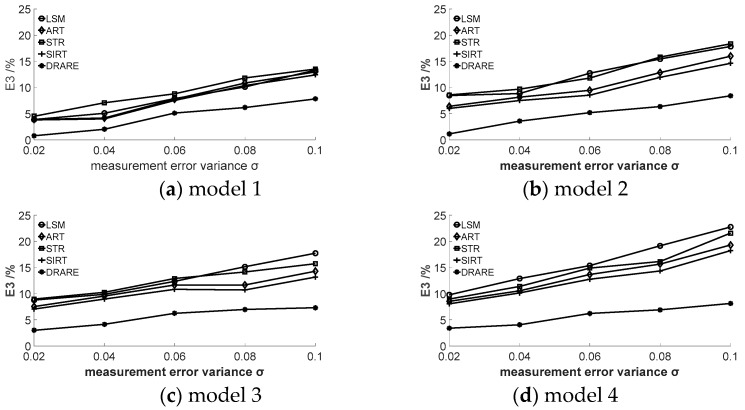
The influence of noise on the mean root square error.

It is an ill-posed inverse problem to reconstruct a temperature field using acoustic tomography where the final solution is sensitive to measurement noise, so a good algorithm should be stable when the TOF data includes noise. The TOF data with noise is used to verify the stability of DRARE in this section. Random noise with standard deviation of 0.02, 0.04, 0.06, 0.08 and 0.10 are added into the simulation TOF data to observe the temperature field change obtained by DRARE. The results of the root mean square (RMS) errors obtained by five algorithms are shown in Figure 9.

It is evident in Figure 9 that each reconstruction algorithm has a certain anti-noise capacity. The anti-noise capacity of SIRT is better than those of LSM, ART and STR. Some stochastic errors are averaged in SIRT since the correction value in each grid cell is the sum of projection errors from all rays passing through the grid cell instead of only concerning one ray as in ART. The anti-noise capacity of DRARE is better than the other four algorithms. Better numerical stability is also obtained with DRARE especially for a complex temperature field reconstruction.

## 6. Conclusions

Acoustic thermometry is a new kind of temperature visualization measurement technology. Accuracy and speed of algorithms play an important role in the reconstruction of temperature field measurements by acoustic tomography. A dynamic model of three-dimensional temperature reconstruction by acoustic tomography is established in this paper. A dynamic algorithm is proposed considering both acoustic measurement information and the dynamic evolution information of the temperature field. An objective function is built which fuses measurement information and the space constraint of the temperature field with its dynamic evolution information. Robust estimation is used to extend the objective function. The method combines a tunneling algorithm and a local minimization technique to solve the objective function. Numerical simulations are performed to evaluate the feasibility and effectiveness of the proposed algorithm with four typical temperature fields. For the cases simulated in this paper, the image quality and noise immunity of the dynamic reconstruction algorithm are better when compared with static algorithms, such as least square method, algebraic reconstruction technique and standard Tikhonov regularization algorithms. Robustness of the dynamic reconstruction algorithm with random noise in the measurement data is analyzed. The results show that the dynamic algorithm has better numerical stability and the advantages are highlighted in the complex temperature field distribution and serious noise. The algorithm can be extended to applications in many areas, such as deep-sea hydrothermal temperature field measurement, temperature field distribution reconstruction in the industrial furnace, atmospheric temperature field distribution measurement, temperature field measurement in the storage grain, temperature field detection inside the microwave heating cavity temperature field, structural health monitoring, and so on.

Twenty sensors are chosen to reconstruct the three-dimensional temperature field considering the limit of cost of acoustic temperature measurement system and the condition in the installation site in this paper. The precision of the reconstruction algorithm is restricted by the limited number of measurement points. It is suggested to increase the number of the sensors and to optimize the installation position of sensors to improve the accuracy of the algorithm. The validation of the experiment is about to be performed in further work.

## Figures and Tables

**Table 1:** Parameters of ART algorithm.

Property	Model 1	Model 2	Model 3	Model 4
relaxation factor	0.24	0.22	0.20	0.20
iteration step	120	120	120	120

**Table 2:** Errors of reconstruction results %.

Algorithm	Model	E1	E2	E3
LSM	model 1	1.02	3.30	1.88
	model 2	7.21	2.45	6.67
	model 3	11.26	6.82	9.86
	model 4	26.05	11.53	10.37
ART	model 1	1.02	3.30	1.88
	model 2	5.41	2.02	5.52
	model 3	7.83	6.23	9.74
	model 4	16.04	10.68	10.34
STR	model 1	1.02	3.30	1.88
	model 2	7.23	2.53	6.69
	model 3	12.37	5.36	9.87
	model 4	26.37	11.50	10.38
SIRT	model 1	1.01	3.30	1.88
	model 2	4.25	1.94	5.21
	model 3	7.04	6.11	7.81
	model 4	15.24	10.13	10.15
DRARE	model 1	0.43	0.89	0.33
	model 2	2.02	1.07	0.41
	model 3	4.85	2.27	2.06
	model 4	8.36	6.15	4.21
